# Unveiling redox mechanism at the iron centers in the mechanochemically activated conversion of CO_2_ in the presence of olivine

**DOI:** 10.1007/s10853-022-06962-x

**Published:** 2022-02-22

**Authors:** Valeria Farina, Maria Domenica Simula, Alessandro Taras, Luca Cappai, Moulay Tahar Sougrati, Gabriele Mulas, Sebastiano Garroni, Stefano Enzo, Lorenzo Stievano

**Affiliations:** 1 Department of Chemistry and Pharmacy, Università Degli Studi Di Sassari and INSTM, Sassari, Italy; 2 ICGM, University Montpellier, CNRS, ENSCM, Montpellier, France

## Abstract

The transformation of olivine during the conversion of CO_2_ to light hydrocarbons activated by mechanochemical treatments at different impact frequencies was studied by a combination of several complementary characterization methods including X-ray diffraction, Raman and ^57^Fe Mössbauer spectroscopy. Several olivine samples were studied as a function of the milling time, indicating the gradual transformation of Fe^II^-containing olivine into new Fe^III^-containing weathering products including iron oxides, magnesium iron carbonates and silicates. The results presented here complement those of a previous study on the weathering process of olivine promoted by mechanochemical activation, by demonstrating the role of the redox activity of the iron species during the activation process. These additional spectroscopic results allow us to thoroughly understand the complex weathering mechanism and to correlate it with the efficiency of the CO_2_ conversion and storage properties of mechanochemically activated olivine.

**Supplementary Information:**

The online version contains supplementary material available at 10.1007/s10853-022-06962-x.

## Introduction

Climate change, together with the biodiversity loss, is one of the biggest challenges facing our world. The key factor believed to induce climate change is the increased global temperature, resulting from the dramatic levels of greenhouse gases emitted in the atmosphere since the advent of the industrial revolution up to nowadays [1]. Carbon dioxide, CO_2_, the most important greenhouse gas, is largely produced by human activities through natural fuels combustions utilized in the electricity production and transportation sector. In the first half of 2020, a considerable reduction of CO_2_ emissions (− 1551 Mt CO_2_) has been recorded, which is substantially due to lockdown measures adopted by many countries to mitigate the COVID-19 pandemic emergency [2]. However, as testified by the increase in July 2020, a rebound of CO_2_ emissions is expected along and beyond 2021 with the risk of exceeding the pre-pandemic levels, thus compromising the ambitious goal of limiting the increase in the mean global temperature to 1.5 °C [3].

Among the tangible solutions proposed by the scientific community, carbon capture, utilization and storage (CCUS) technologies have been indicated as one of the most promising methods for the effective mitigation of carbon dioxide in the atmosphere [3]. These processes typically involve the sequestration of CO_2_ into specific sites such as geological reservoirs and, alternatively, chemical transformation into inorganic minerals (mineral carbonation) or added values chemicals and renewable fuels, to finally generate a virtuous CO_2_ cycle [4]. Mineral weathering is probably the main natural process observed in our planet for recycling carbon dioxide: It is well known, in fact, that more than 100 million tons of carbon per year are sequestrated through silicate weathering [5]. In this regard, minerals with a high magnesium content such as serpentine and olivine represent the best candidates for this application [6, 7]. Olivine is one of the most abundant minerals on Earth: It constitutes mafic and ultramafic igneous rocks covering more than 84% in volume of the terrestrial mantle. Olivine minerals consist of a solid solution of fayalite (Fa, Fe_2_SiO_4_) and forsterite (Fo, Mg_2_SiO_4_), in different ratios. The hydration of olivine leads to the formation of secondary minerals, via a process called “serpentinization,” a widespread phenomenon on Earth and Mars mantles occurring generally at temperatures around 300 °C [8–11]. During this process, Fe- and Mg-based silicate minerals react with water producing H_2_ and minerals of the serpentine group [(Mg, Fe)_3_Si_2_O_5_(OH)_4_], as described in Eq. 1:

1 $$6 \, \left( {{\text{Mg}},{\text{Fe}}} \right)_{2} {\text{SiO}}_{4} + \, 7{\text{ H}}_{2} {\text{O}} \to 3 \, \left( {{\text{Mg}},{\text{Fe}}} \right)_{3} {\text{Si}}_{2} {\text{O}}_{5} \left( {{\text{OH}}} \right)_{4} + {\text{ Fe}}_{3} {\text{O}}_{4} + \, H_{2}$$

This process results in the partial oxidation of the ferrous iron of olivine which is transformed into ferric iron in magnetite (Fe_3_O_4_) and other minerals, going along with the simultaneous reduction of water to molecular hydrogen. This redox reaction is exothermic and leads to the formation of reducing fluids rich in hydrogen.

The latter may react with CO_2_ through a Sabatier or Fischer–Tropsch-type (FTT) mechanism forming CH_4_ and light hydrocarbons [9], while, at the same time, part of the CO_2_ can be fixed in the form of carbonates. Such serpentinization process in the presence of CO_2_ can thus be described by the following reactions (Eqs. 2, 3):

2 $$\left( {{\text{Mg}},{\text{ Fe}}} \right)_{2} {\text{SiO}}_{4} + \, n{\text{H}}_{2} {\text{O }} + {\text{ CO}}_{2} \to \, \left( {{\text{Mg}},{\text{ Fe}}} \right)_{3} {\text{Si}}_{2} {\text{O}}_{5} \left( {{\text{OH}}} \right)_{4} + {\text{ Fe}}_{3} {\text{O}}_{4} + {\text{ CH}}_{4}$$

3 $$\left( {{\text{Mg}},{\text{ Fe}}} \right)_{2} {\text{SiO}}_{4} + \, n{\text{H}}_{2} {\text{O }} + {\text{ CO}}_{2} \to \, \left( {{\text{Mg}},{\text{ Fe}}} \right)_{3} {\text{Si}}_{2} {\text{O}}_{5} \left( {{\text{OH}}} \right)_{4} + {\text{ Fe}}_{3} {\text{O}}_{4} + {\text{ MgCO}}_{3} + {\text{ SiO}}_{2}$$

Although these processes are thermodynamically favored, their reaction rates are very slow and under thermal conditions their performance is not suitable for practical purposes. Kinetic improvements are possible by pre-activating the powders through mechanical processing [12–19]. Recently, both the processes (reactions 2 and 3) have been extensively investigated under mechanochemical activation for the first time [20–22]. Interestingly, if on the one hand the production of hydrogen and light hydrocarbons (mainly methane) is strongly improved through ball milling with respect to thermal activation, on the other hand the competitive carbonation rate can be enhanced for increasing milling times under dry and wet conditions. This evidence makes the mechanically activated reaction of particular interest, being it able to simultaneously generate added value products such as carbonates (building and thermal storage materials) and light hydrocarbons (renewable chemical fuels). However, if the formation of magnesite-based system is quite understood [21], a clear description of the redox mechanism, crucial for the catalysis of the hydrogen production and FTT processes, is still lacking [22].

In this work, the solid products obtained during and upon the olivine weathering reaction with carbon dioxide under mechanical treatment were characterized via a multi-technique approach including complementary analytical methods in order to clarify the role played by the iron species and shed new light on the redox mechanism of this complex process.

## Methods

Natural olivine was supplied by Satef-HA (Italy). Average composition expressed as weight % of oxide of each element contained in the material was provided by the supplier: 50.00% MgO, 41.50% SiO_2_, 7.30% Fe2O_3_, 0.29% Cr_2_O_3_, 0.40% Al_2_O_3_, 0.30% NiO, 0.10% MnO and 0.10% CaO. For more details, see the Supporting information section (Table S1).

Mechanochemical activation was carried out using a Spex Mixer Mill mod. 8000, as previously described in detail in ref. [20]. Four grams of the pristine olivine powder and 0.6 ml of deionized H_2_O were ball-milled in the presence of CO_2_ varying the rotation speed at 750, 875 and 100 rpm for increasing time using a stainless steel jar (76 cm^3^) and three stainless steel balls of 3.80 g each. Two valves set on the top and bottom of the milling jar allowed both the connection to the gas reservoir (CO_2_) and the analysis of the gases produced upon mechanochemical activation. Before the mechanochemical treatment and the introduction of CO_2_, the jar was degassed by applying a dynamic vacuum of 10^−3^ mbar for 10 min. Before any experiment, in order to remove carbon contamination, powders were washed with acetone for 5 min in an ultrasonic bath and then dried at 433 K under dynamic vacuum for 1 h.

Gas samples were analyzed by means of gas chromatography with a PerkinElmer 8600 (Wide-bore column GSQ 115-3432-J&W Scientific and FID detector) and a Fisons 8000 (molecular sieves packed column and an HWD detector). Standards for the quantitative analysis of CH_4_ and hydrocarbons (0.1 and 1% v/v) and high-purity carbon dioxide gas (N5—99.999%; CH_4_ b 1 ppm) were provided by Sapio (Italy).

Structural characterization of the materials was performed using a Rigaku SmartLab X-ray Diffractometer (XRD) with a Bragg–Brentano geometry, Cu K_α_ radiation = 1.54178 Å) and a graphite monochromator in the diffracted beam. Semiquantitative evaluation of phase abundance and structural features were obtained for all the XRD patterns, by nonlinear least-square refinement procedure, according to Rietveld method, and using the MAUD (Materials Analysis Using Diffraction) software [23].

Raman spectroscopy measurements were performed at ambient temperature, using a HORIBA Jobin Yvon LabRAM ARAMIS spectrometer, equipped with a blue diode laser (D473, *λ* ≈ 473 nm). The laser beam was focused using a 50 × objective. Given the small size of the Raman probe (about 5 μm), spectra of the studied samples were collected twice in different areas to identify any inhomogeneity, with a scan time of 120–240 s. Experimental data were analyzed comparing peaks of mineral standards from the RRUFF database, using the program CrystalSleuth [24, 25].

^57^Fe Mössbauer spectra were measured at ambient temperature and for specific samples at 5 K. A source of ^57^Co/Rh, always kept at room temperature, was used, and measurement times ranged from 24 to 48 h. Isomer shifts are given relative to α-Fe metal at room temperature. The spectra were fitted with appropriate combinations of Lorentzian line shapes using the PC-Mos II computer program [26]. In this way, typical hyperfine parameters such as the isomer shift (IS), the electric quadrupole splitting (QS), the linewidth at half maximum (LW) and the relative absorption area of the different spectral components were determined.

## Results and discussion

### Mechanical Activation and gases products

The crystallographic characterization of the powders by Rietveld refinement of the XRD patterns was already described in detail in ref. [20]. Pristine olivine consists of three phases: forsterite (Fe_0.1_Mg_1.9_SiO_4_) 90.8 wt.%, enstatite ferroan (Fe_0.3_Mg_1.7_Si_2_O_6_) 7.5 wt% and clinochlore (Al_1.84_Fe_0.5_H_8_Mg_4.5_O_18_Si_3.16_) 1.7 wt.%. In summary, the pristine material is a Mg-rich olivine with an approximate composition Fo_90_Fa_10_.

**Figure 1:**
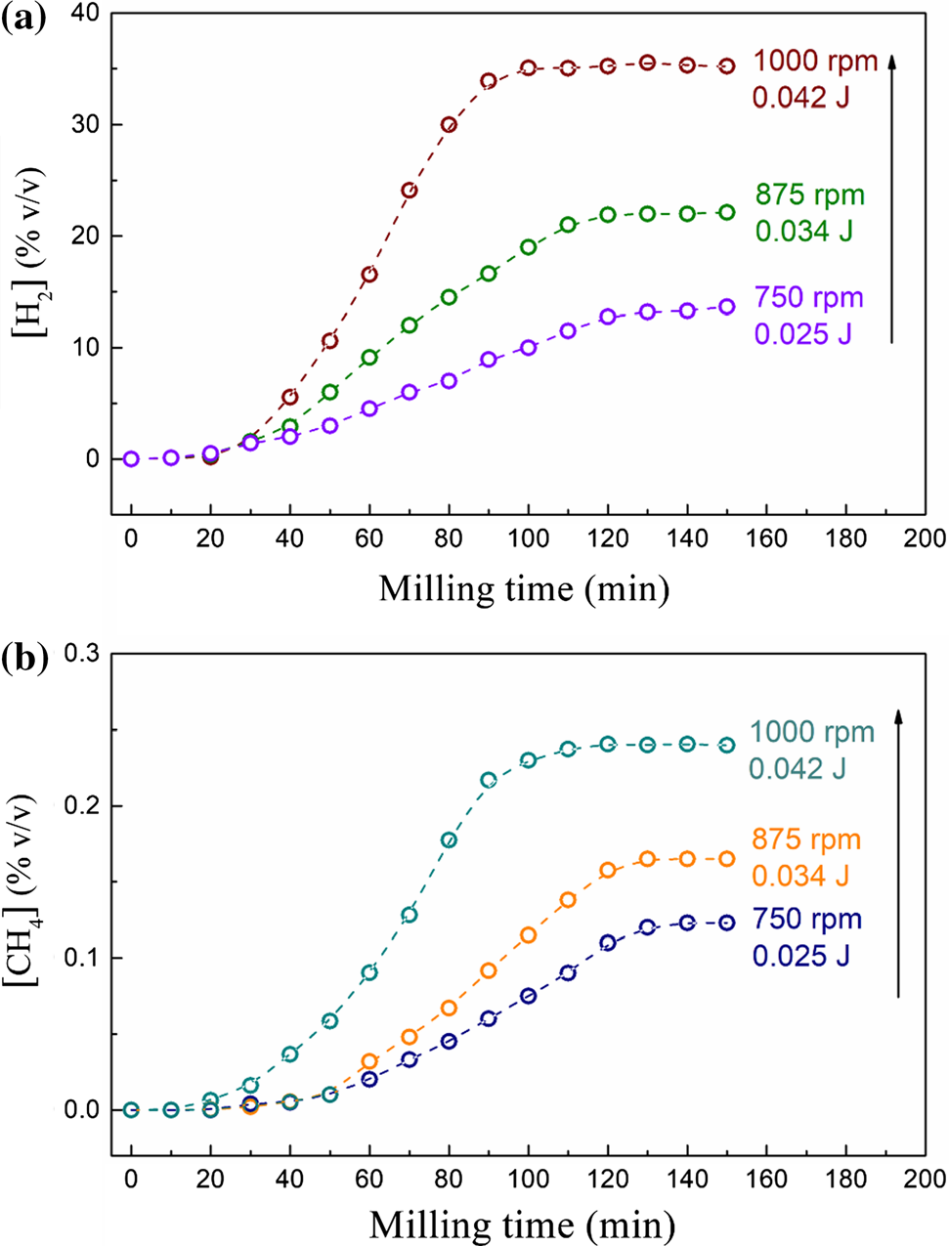
Hydrogen (**a**) and methane (**b**) concentration as a function of milling time, generated using a milling velocity of 750, 875 and 1000 rpm. The corresponding energy impact [J/collision], evaluated according to [27], is also inserted in both panels

As a first experiment, the wet olivine powders were exposed to a carbon dioxide flow (10 ml/min) into a static reactor for 60 min, and no conversion was observed by GC analysis. Similarly, negligible amounts of hydrogen and methane were recorded when the pristine powders were subjected to simply agitation at 875 rpm without grinding bodies inside the reactor. Conversely, when the reaction was conducted under mechanical activation, hydrogen and methane were both detected. Figure 1 shows the concentration of H_2_ (Fig. 1a) and CH_4_ (Fig. 1b) generated as a function of the milling time at different milling velocities (from 750 to 1000 rpm). After an induction period of 20 min, hydrogen is generated according to Eq. 1, with a linear increase, indicating the highest reaction rate, before reaching a plateau between 100 and 120 min of milling time. A similar trend is observed for methane production, even though with a lower concentration (up to 0.25% v/v) with respect to hydrogen (up to 35% v/v). Also, the impact energy significantly affects both hydrogen and methane production rates. In fact, the linear slope of the concentrations reported in Fig. 1 becomes increasingly steeper as the impact energy and the collision frequency increased.

These results suggest that the production of hydrogen and methane obtained in this specific reaction under mechanical activation probably requires a minimum impact energy: In fact, as mentioned above, the absence of grinding media and mixing did not induce any chemical reaction involving the conversion of carbon dioxide and water. Further experiments are necessary to determine the impact energy threshold required to activate the reaction, but, in according to previous studies, the activation energy under mechanical input observed in such experiments is lower than that which was registered as necessary for triggering a FFT process catalyzed by Fe-based oxide, which corresponds to 85 kJ/mol [22]. It is important to highlight that mechanochemical effects can be measured, and the results achieved at different impact energy corroborate the important enhancement on the reaction kinetics with respect to the thermally activated one. To gain more knowledge, its results very important to define the products and side products formed during the reaction which can play the role of catalysts in this mechanically activated carbon dioxide transformation process.

**Figure 2:**
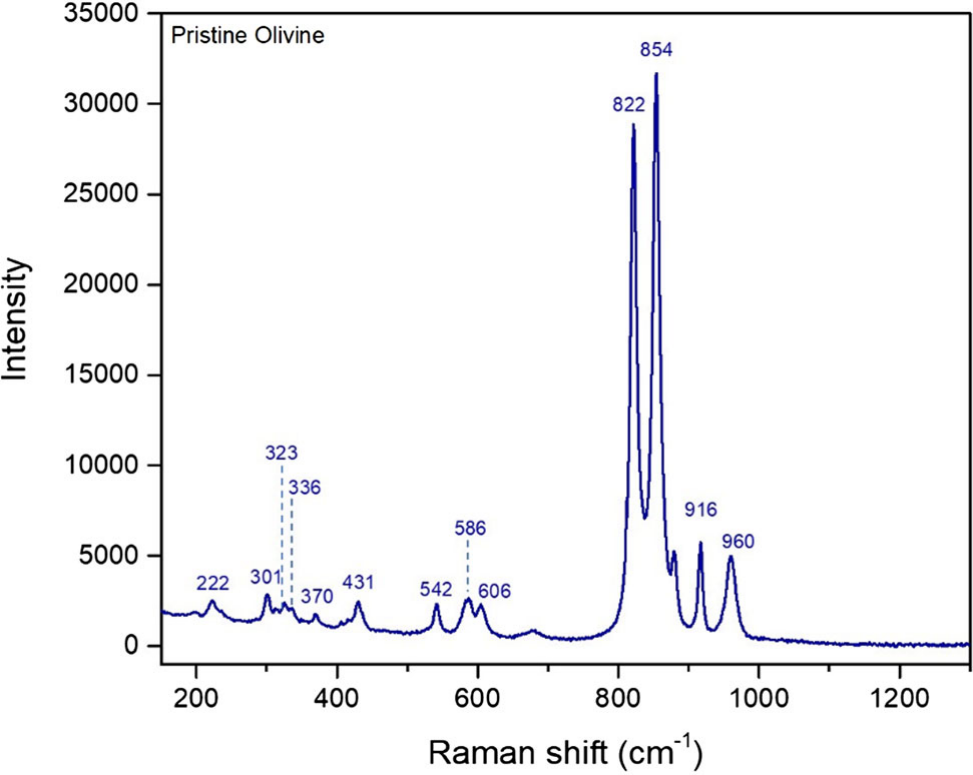
Raman spectrum of pristine olivine (Fo_90_Fa_10_)

To determine the evolution of the forsterite-based species, Raman spectroscopy was applied because it is relatively sensitive to the material surface, in particular with respect to X-ray diffraction used in previous works [22]. The Raman spectrum of pristine olivine is shown in Fig. 2. From a direct comparison with literature data [24, 25], all the observed Raman shifts are close to those of forsterite, as expected given the high Mg content of the material (cf. Table S1).

**Figure 3:**
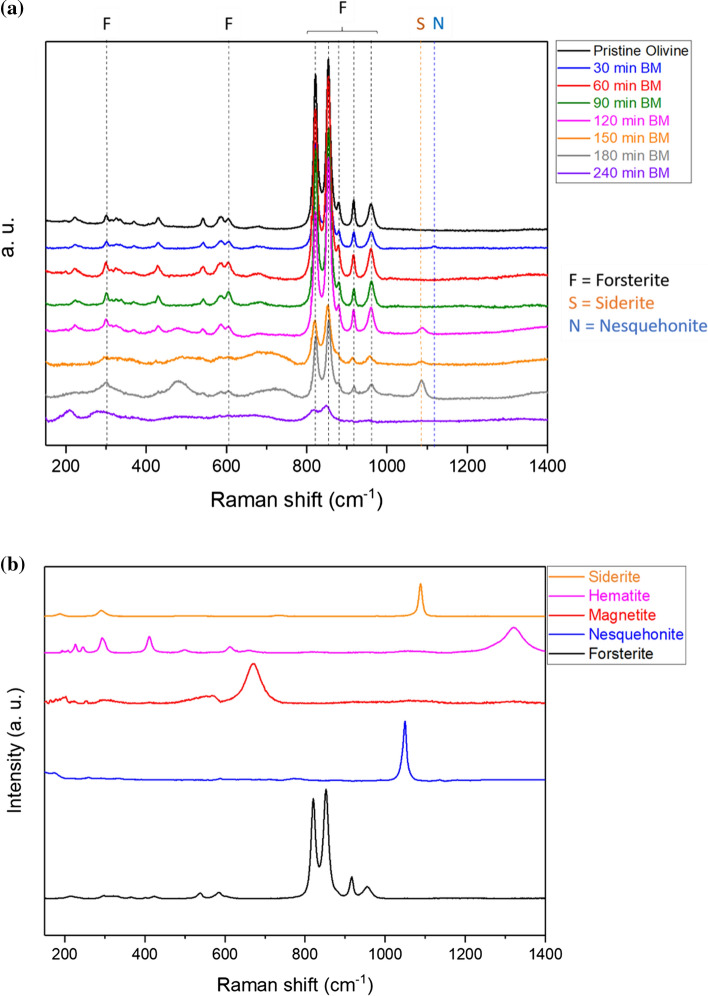
(**a**) Raman spectra measured at increasing milling times. Notes: F = forsterite, S = siderite. (**b**) Raman spectra of known standards

The band at 222 cm^−1^ is related to the translation modes of (SiO_4_) and to the M2 site (M represents a metal cation. Cf. SI, Figure S1). The bands in the 301–370 cm^−1^ region are assigned to the vibration modes of (SiO_4_) and M2 site. The bands from 431 to 606 cm^−1^ represent SiO_2_ bending modes, and those from 822 to 960 cm^−1^ the SiO_2_ stretching ones. Further analyses were then performed on the solid products recovered upon ball milling (impact energy 0.034 J) at selected times. Figure 3a shows the Raman spectra acquired on olivine samples ball-milled in the 0–240 min range and the known standards from RRUFF database (Fig. 3b), for a direct comparison [24, 25]. The dominating phase for all ball-milled samples is forsterite, with the main bands located at 323, 610 and 970 cm^−1^. At increasing milling times, the mineralogy changes significantly: Starting from 120 min of milling, broad bands appear in the Raman spectra at 300, 480 and 700 cm^−1^, probably related to the presence of ill-defined Fe^3+^ oxide species. In fact, the typical bands of trivalent iron oxides usually show up at similar wavelengths. In the samples milled for 120, 150 and 180 min, the presence of a further band at 1088 cm^−1^ indicates the presence of siderite, a common Fe-based carbonate. All peak intensities associated with the above-mentioned phases decreased with increasing milling times, which can be attributed to the decrease in the particle size and/or partial amorphization of the powders subjected to prolonged mechanical treatment. The emphasized broadening of the siderite peak, in particular, does not allow its observation when the milling time exceeds 240 min (Fig. 3a). These features are better highlighted in Figure S2.

**Figure 4:**
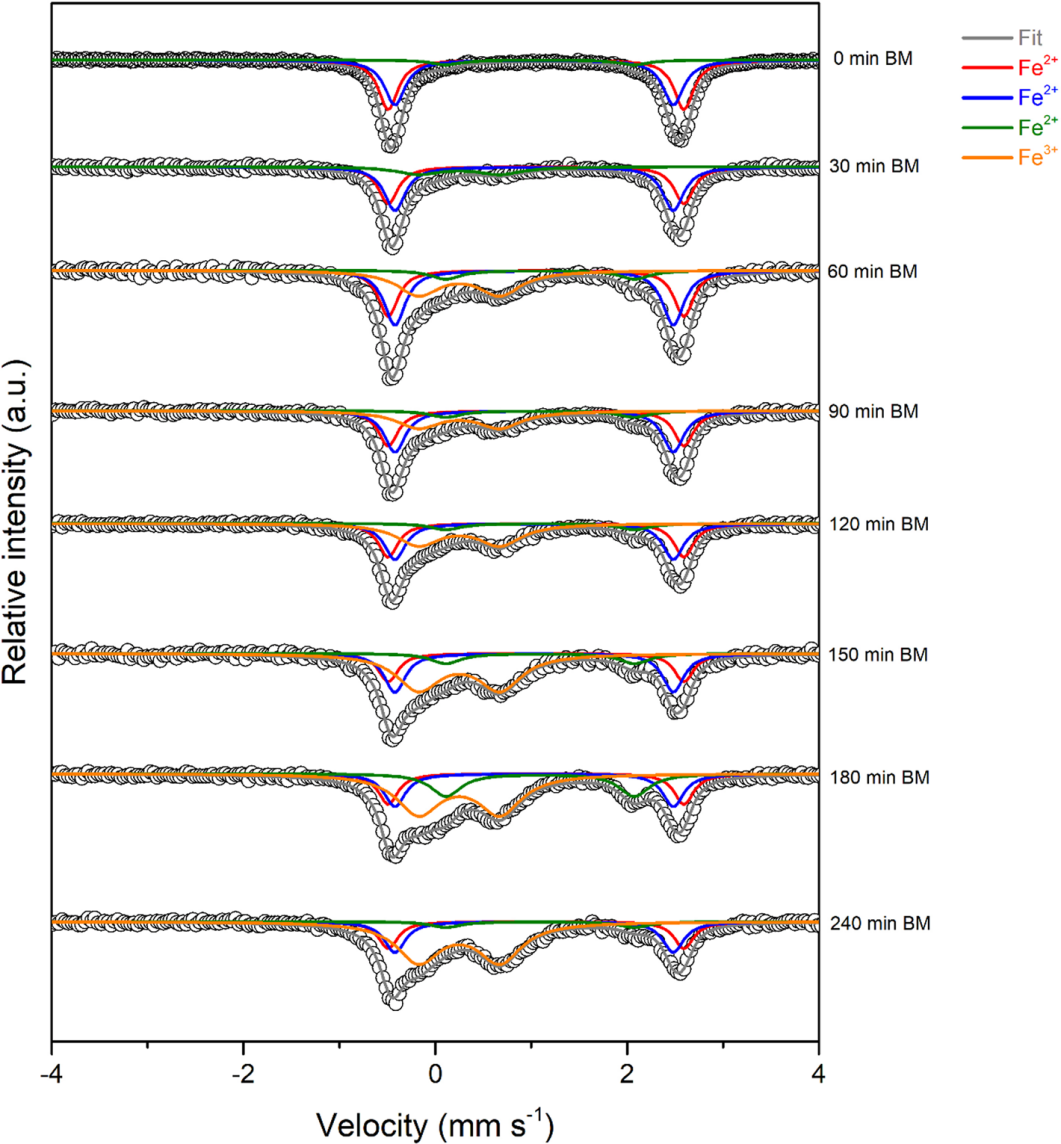
Mössbauer spectra measured at room temperature of all the samples, plotted for increasing milling time. Olivine site M1: red line; olivine site M2: blue line; pyroxene: green line; Fe III: orange line

Mössbauer spectra (Fig. 4) were measured at increasing milling times to verify the evolution of the oxidation state of the iron species. The Mössbauer spectra of pristine olivine (indicated as 0 min BM in Fig. 4) show an asymmetric quadrupole doublet which can be fitted by using at least three independent spectral components. Comparing the experimental values of isomer shift, IS, and quadrupole splitting, QS (Table 1), with those reported in the literature [28–32], it is easy to attribute the two doublets with high intensities (red and blue lines in Fig. 3) to Fe^2+^ located at M1 and M2 sites, respectively, confirming that also in this case, there is no cationic site preference for the iron ions. The third doublet (green line), less intense than the other two, is characteristic of octahedral Fe^2+^ in pyroxene [(Mg,Fe)Si_2_O_6_] [29]. Such results agree with the composition determined by XRD analysis, which detected the presence of forsterite, enstatite ferroan and clinochlore [20]. The whole series of spectra measured at increasing milling times (Fig. 4) could be fitted simultaneously using four quadrupole doublets with common hyperfine parameters. The results of this simultaneous fit are summarized in Table 1.

**Table 1:** Mössbauer parameters for each component presented in Fig. 4

	QS (mms^−1^)	IS (mms^−1^)	LW (mms^−1^)	Fe type
Olivine site M1	3.08 ± .001	1.15 ± 0.00	0.25 ± 0.01	Fe^2+^
Olivine site M2	2.90 ± 0.01	1.13 ± 0.00	0.24 ± 0.01	Fe^2+^
Pyroxene (Mg,Fe)Si_2_O_6_	1.96 ± 0.01	1.19 ± 0.00	0.38 ± 0.01	Fe^2+^
Fe(III) oxides	0.85 ± 0.01	0.35 ± 0.00	0.54 ± 0.00	Fe^3+^

**Figure 5:**
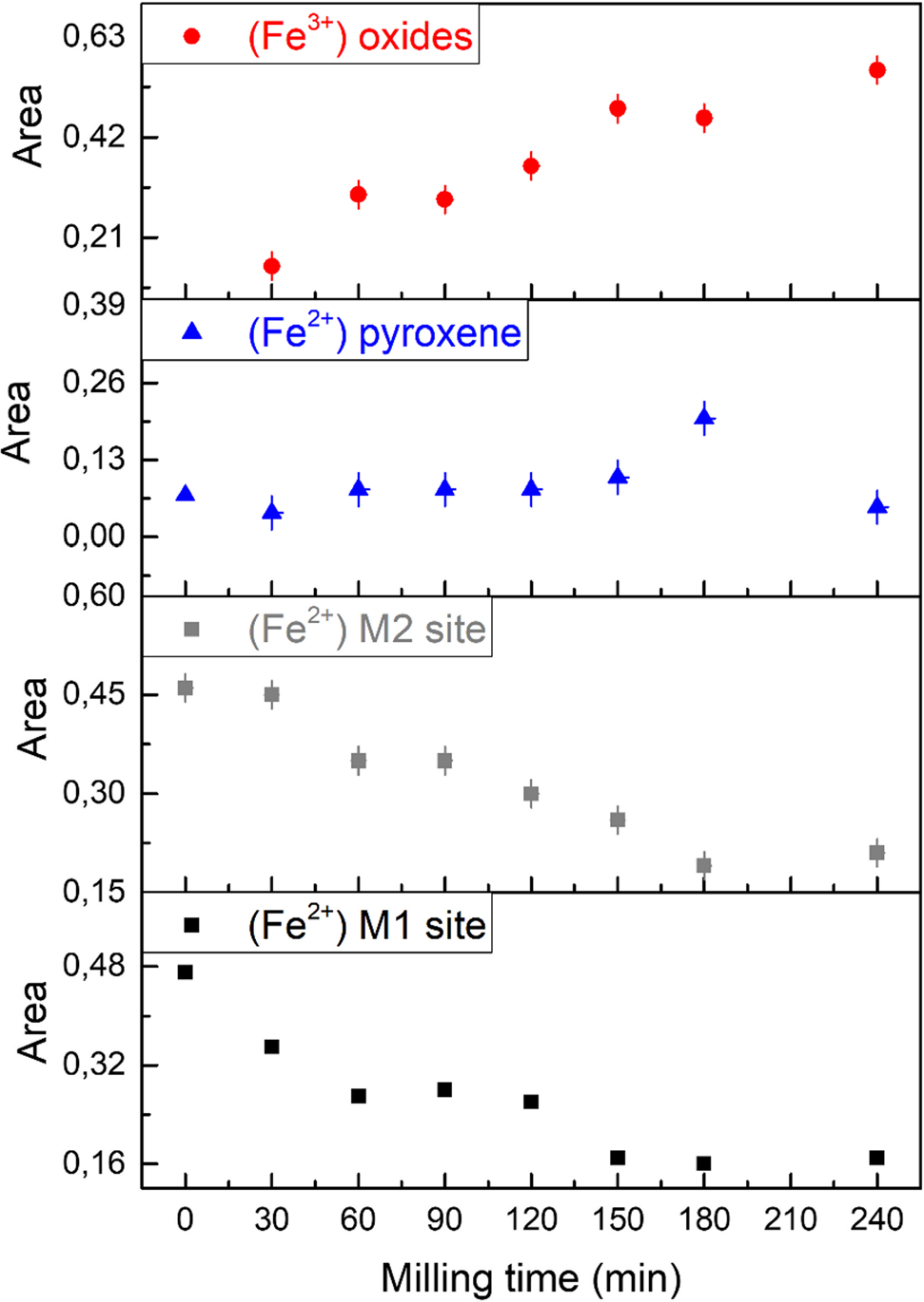
Relative area of each doublet for all the samples, plotted as a function of the milling time

In this series of spectra, the first three doublets correspond to those of pristine olivine, whereas the additional fourth doublet is typical of trivalent iron with hyperfine parameters in the typical range of disordered and/or superparamagnetic iron oxides/oxyhydroxides. This doublet appears already after 30 min of milling, indicating that the oxidation of Fe^II^ to Fe^III^ occurs in the first step of the reaction. The fraction of iron ions in the higher oxidation state increases with increasing milling time, as confirmed by the amplified relative resonance area of the Fe^3+^ doublet (Fig. 5, Table S1). These results allow us to validate the preliminary hypothesis, according to which the hydrogen evolution during the CO_2_ conversion to hydrocarbons is the proof of the occurrence of a serpentinization-like process. Moreover, the observed formation of Fe^3+^ agrees well with the observations of Raman spectroscopy. The sample measured after 150 min of milling is characterized by the four doublets described previously attributed to Fe^2+^ in the two sites of olivine, pyroxene site and superparamagnetic Fe^3+^ oxides [28, 29]. As the relative area of Fe^3+^ doublet increases, those of Fe^2+^ sites related to pristine olivine decrease, while the relative area of Fe^2+^ related to the pyroxene site remains almost unchanged up to 180 min. After that, it varies slightly, indicating a possible participation of this phase to the reaction only after long milling times. Hence, even though Fe^II^ is consumed during the process, at 240 min a small amount is still available, as Fe^II^ both in M1 and M2 sites of olivine and in pyroxene. In principle, it is then expected that CO_2_ conversion to hydrocarbons might thus continue for prolonged milling times.

**Figure 6:**
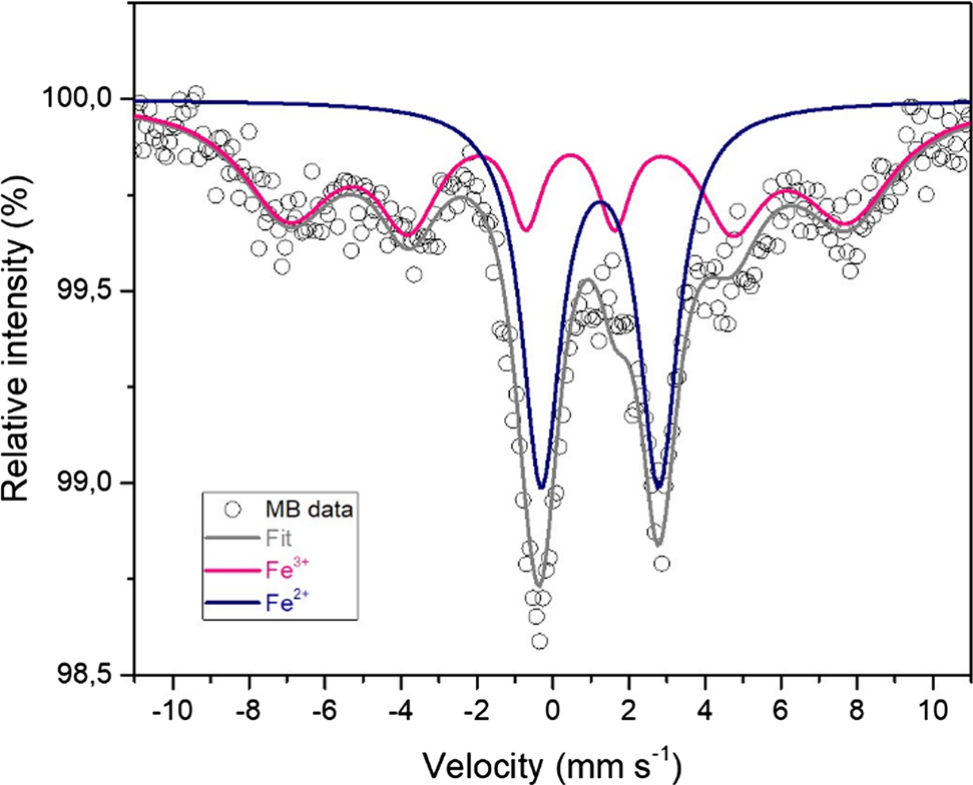
^57^Fe Mössbauer spectra recorded at 5 K, of the sample milled for 150 min

In order to better understand the nature of the iron oxide species involved in the reaction, a Mössbauer spectrum of the sample milled for 150 min was also recorded at 5 K (Fig. 6).

**Figure 7:**
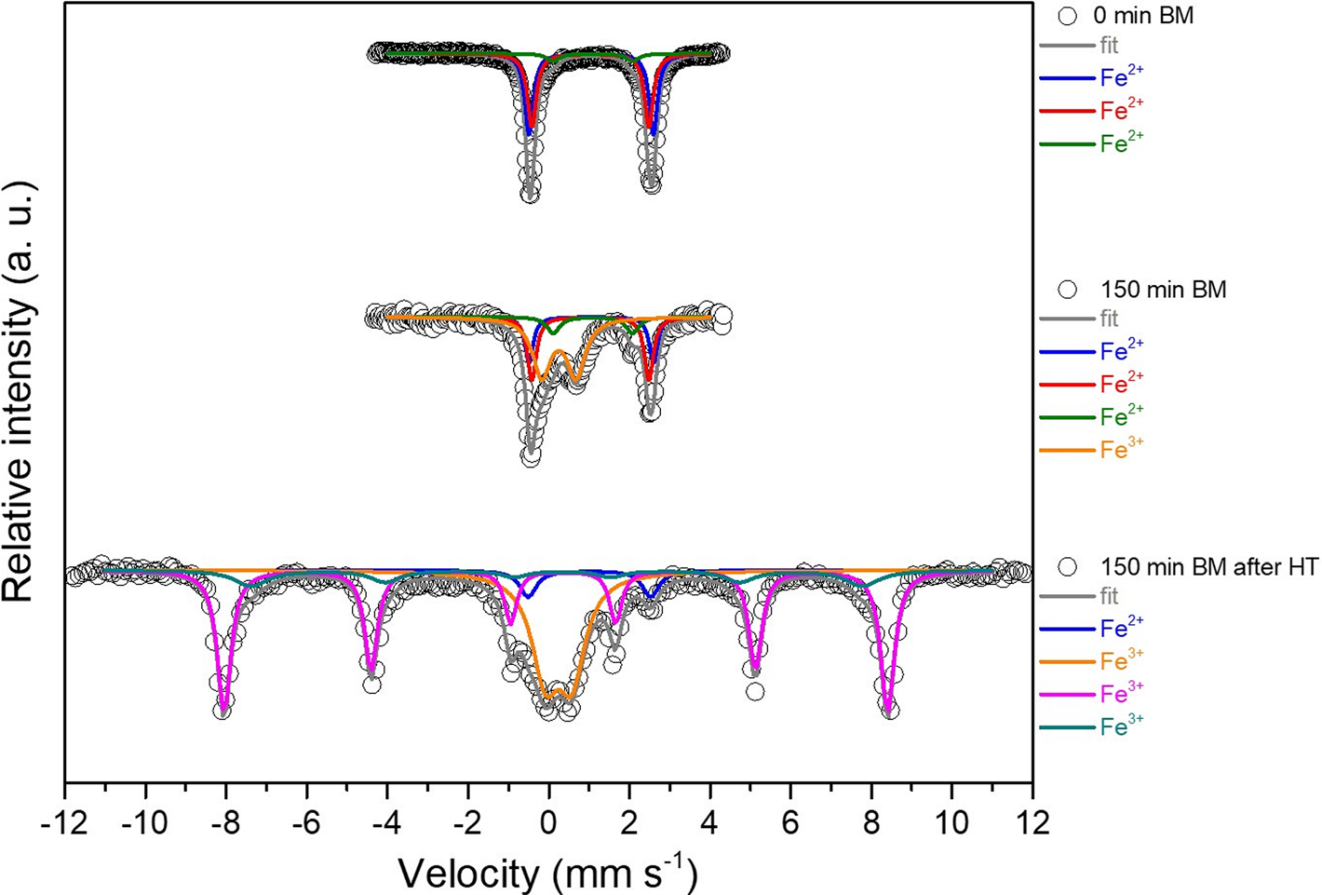
Comparison between the Mössbauer spectra of the sample milled for 150 min and thermally treated at 1273 K. Pristine olivine and 150-min ball-milled sample spectra are included for simple comparison. HT = heat treatment

In this spectrum, in addition to the olivine doublets, which retain their shape, the Fe^III^ quadrupole doublet is replaced by a broad magnetic sextet, with IS = 0.55 mm s^−1^ and QS = – 0.13 mm s^−1^. Such modification of the spectrum at low temperature is characteristic of the presence of disordered or nanosized Fe^III^ oxides undergoing superparamagnetic relaxation, thus showing magnetic order only below the so-called blocking temperature. The sample milled for 150 min was then thermally treated at 1273 K under Ar atmosphere and further characterized via Mössbauer spectroscopy. As depicted in Fig. 7, the corresponding Mössbauer spectrum includes two additional magnetic sextets with respect to the untreated sample.

The first sextet (violet line) has hyperfine parameters (IS = 0.38 mm s^−1^, QS = – 0.18 mm s^−1^ and hyperfine field B = 51.01 T) typical of crystalline hematite at room temperature [33]. The parameters of the second sextet (dark cyan line, IS = 0.27 mm s^−1^, QS = – 0.14 mm s^−1^ and B = 47.0 T) could be related to the presence of less well-crystallized hematite. However, the possible substitution of Fe with Mg cations in the structure might also cause a decrease in the magnetic hyperfine field value. Finally, the formation of minor amounts of magnetite (or maghemite, *γ*-Fe_2_O_3_) cannot be completely excluded. As already described in ref. [20], the presence of both magnetite and hematite in the sample heated at 1273 K was observed. Maghemite and magnetite, in fact, share the same inverse spinel crystal structure and form a solid solution in the whole composition domain and differ only in the oxidation state of the iron centers. The absence of the sextet typical of mixed divalent/trivalent iron of magnetite indicates the complete oxidation of the iron to Fe^III^, and thus the presence of maghemite [28]. This additional experiment confirms their formation already after short milling times. The additional thermal treatment performed on the powders favors the grain growth and then the crystallization of these oxides, making them easily detectable through Mossbauer analyses conducted at room temperature.

From these results, it is possible to establish the manner in which Fe is partitioned among the reaction products: Under these mechanochemical conditions, the weathering reaction of pristine olivine constituted by a blend of fayalite–forsterite [(Mg_1.80_Fe_0.20_)SiO_4_] and ferroan enstatite [(Mg,Fe)Si_2_O_6_] results in the gradual extraction of the divalent iron. The oxidation of the iron into Fe^III^, forming partially disordered-containing iron oxides identified by ^57^Fe Mössbauer spectroscopy as a mixture of hematite and maghemite/magnetite, is accompanied by the reduction of water protons to produce a significant amount of molecular hydrogen. The presence of serpentine minerals with general formula [(Mg,Fe)_3_Si_2_O_5_(OH)_4_] was not confirmed, corroborating previous studies on olivine weathering reactions conducted under mechanical processing [20–23]. This represents a significant difference with the hydrothermal pathways reported in the literature [34]. A first stage of the mechanochemical mechanism can be then summarized by the following reaction:

4 $$18{\text{ Mg}}_{1.8} {\text{Fe}}_{0.2} {\text{SiO}}_{4} + 19{\text{ H}}_{2} {\text{O}}_{{}} + 16{\text{CO}}_{2} \to {\text{ Fe}}_{2} {\text{O}}_{3} + 19H_{2} + \, 18{\text{MgSiO}}_{4} + \, 16{\text{ Mg}}_{0.9} {\text{Fe}}_{0.1} {\text{CO}}_{3}$$

As evidenced by reaction 4, no hydrocarbons are included in the products because they are generated by the direct reaction between gaseous hydrogen and carbon dioxide, which take place once the formation of Fe^II^ oxide species has occurred. The iron oxide species catalyze the formation of light hydrocarbons by the proposed FFT-type process [35, 36]. A further source of methane could be the thermodynamically favored reaction of the carbonates with hydrogen. However, the as-expected magnesium oxide was not detected in any steps of the weathering reaction. This agrees with the fact that the hydrogenation of carbon dioxide results favored in the temperature range of 273–573 K as emerged from the ΔG profiles reported in Figure S3. Therefore, it is possible to state that, if carbonatation is competing, under certain conditions, with the generation of hydrocarbons, the latter process can be only reconducted to the reaction of hydrogen with gaseous carbon dioxide catalyzed by the iron oxides species here determined.

We are therefore convinced that the mechanically activated weathering reaction can potentially open a new season for developing a valid approach, easy scalable to the industrial level, to include carbon dioxide into a virtuous cycle. Furthermore, the comprehension of the aspects at single impact event can be important for getting light into the abiotic synthesis of simple organic compounds related with the origin of life [37]. For these purposes, the systematic quantification of mechanochemical effects and the exploration of potential amorphous species could lead original achievements in the investigation of this field.

## Conclusion

In this work, the weathering reaction of olivine in the presence of carbon dioxide was investigated under mechanical processing varying the milling parameters. The evolution of hydrogen and light hydrocarbons started after an induction period and then reached a maximum of conversion increasing as the impact energy and impact frequency increased. The total amount of gases generates also depends on the energy provided, at the local scale, to the portion of collided powders. Raman and ^57^Fe Mössbauer spectroscopy are proved to be useful techniques, complementary to X-ray diffraction, for defining the mechanics behind the mechanically activate weathering reaction. These techniques allowed evidencing the occurring of a *Serpentinization*-like process, *i.e.*, the oxidation of olivine’s Fe^II^ to Fe^III^ oxides during the production of hydrogen and the CO_2_ conversion to light hydrocarbons activated by mechanical treatment. Despite the remarkable evolution of H_2_, however, no presence of serpentine mineral could be evidenced on the sample powders to confirm the occurrence of the process. Most probably, the high collision rate induces the gradual extraction of the divalent iron from the pristine olivine, forming a mixture of ill-defined nanosized iron oxides similar to hematite and maghemite/magnetite as shown by ^57^Fe Mössbauer spectroscopy. These results underline the difference between the weathering reaction processes of olivine observed under mechanical or a more conventional activation process (*i.e.*, hydrothermal). This aspect could represent the key for understanding the remarkable production of gases under mechanochemical conditions.

## Supplementary Information

Below is the link to the electronic supplementary material.

Supplementary file1 (DOCX 829 kb)
