# Supplementary material for: Unveiling redox mechanism at the iron centers in the mechanochemically activated conversion of CO2 in the presence of olivine
Source: J Mater Sci. 2022 Feb 22;57(22):10017–27. doi: 10.1007/s10853-022-06962-x (PMC8863097; doi:10.1007/s10853-022-06962-x)
Supplement: Supplementary file 1 — Supplementary file1 (DOCX 829 kb) [file 10853_2022_6962_MOESM1_ESM.docx]

Supplementary information

Unveiling redox mechanism at the iron centres in the mechanochemically activated conversion of CO_2_ in the presence of olivine.

Valeria Farina^1^, Maria Domenica Simula^1^, Alessandro Taras^1^, Luca Cappai^1^, Moulay Tahar Sougrati^2^, Gabriele Mulas^1^, Sebastiano Garroni^1*^, Stefano Enzo^1^, Lorenzo Stievano^2^

^1^ Department of Chemistry and Pharmacy, Università degli Studi di Sassari and INSTM, Sassari, Italy.

^2^ ICGM, Univ. Montpellier, CNRS, ENSCM, Montpellier, France.

Composition of pristine olivine

The composition of the pristine commercial olivine was provided by the supplier (Satef) and it is reported in Table S1. The same composition was also checked by EDX-SEM by averaging the analysis over several large representative areas of the sample as well as by XRF on the pristine powder. A quite good correspondence between the two analyses and the data provided by the supplier can be observed.

Table S1. Composition of the pristine olivine provided by the supplier and measured by SEM-EDX and XRF.

| Element | Elemental content | | | |
| --- | --- | --- | --- | --- |
|  | **Supplier (%)** | **SEM-EDX (%)** | **XRF (%)** | **Rietveld (%)** |
| Mg | 30.15 | 23.0 – 29.5 | 30.1 | 31.7 |
| Si | 19.38 | 15.9 – 16.5 | 20.5 | 19.3 |
| Fe | 5.11 | 4.7 – 6.3 | 9.2 | 4.2 |
| Cr | 0.20 | - | 0.4 | - |
| Al | 0.21 | 2.4 – 4.9 | 0.4 | 0.2 |
| Ni | 0.24 | - | 0.6 | - |
| Mn | 0.08 | - | 0.1 | - |
| Ca | 0.07 | - | 0.2 | - |
| O | 44.55 | 45.9 – 47.8 |  | 44.3 |

These compositional values can be compared to those calculated from the phase compositions obtained by quantitative Rietveld refinement of the diffraction pattern of pristine olivine, achieved using the following refined stoichiometries for the different crystalline phases detected in the pattern: Forsterite ferroan (Mg_1.9_Fe_0.1_)SiO_4_, Enstatite, (Mg_1.7_Fe_0.3_)Si_2_O6; Clinochlore, Al_1.84_Fe_0.5_H_8_Mg_4.5_O_18_Si_3.16_. A very good agreement between these values and those of the different chemical analyses can be observed.

Olivine crystal structure

Orthorhombic olivine hosts the bivalent cations, Fe^2+^ and Mg^2+^, in the octahedral sites (Figure S1 – orange color), coordinated with six neighboring oxygen atoms of SiO^4-^ tetrahedra (Figure S1 – blue clor). Two octahedral sites can be distinguished: one with smaller and regular size (called **M1**) typically occupied by Fe^2+^ and another type (called **M2**) occupied by Mg^2+^.


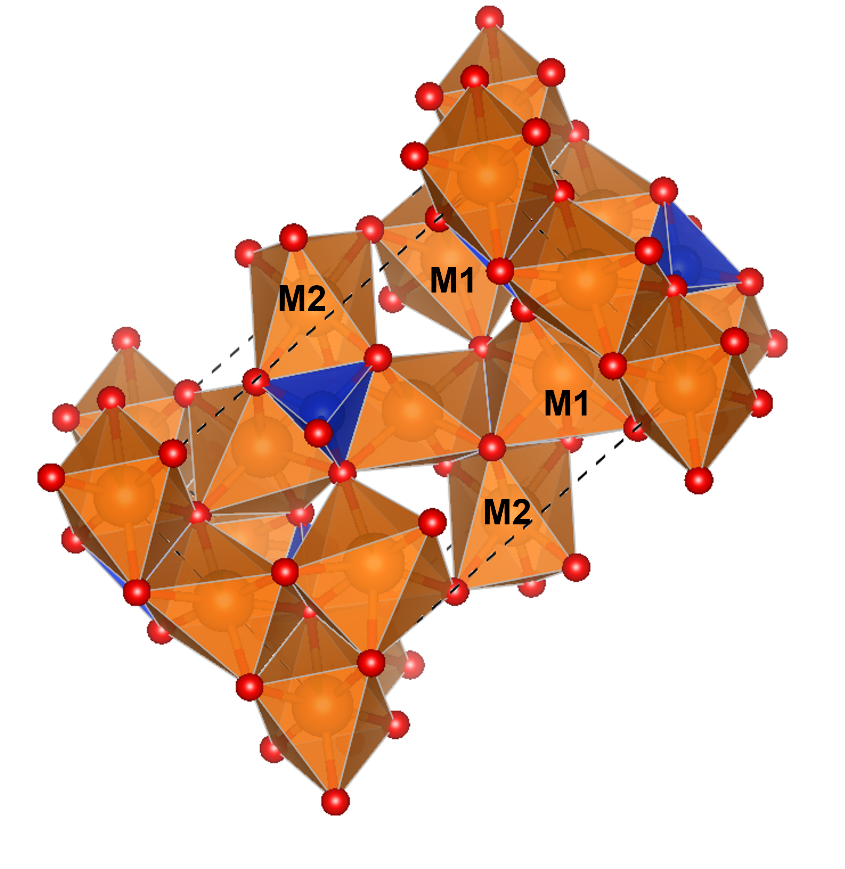


Figure S1. Representative arrangement of tetrahedral and octahedral sites in olivine showing the M1 and M2 sites. The unit cell has been designed by VESTA software [1]

Raman spectra

*
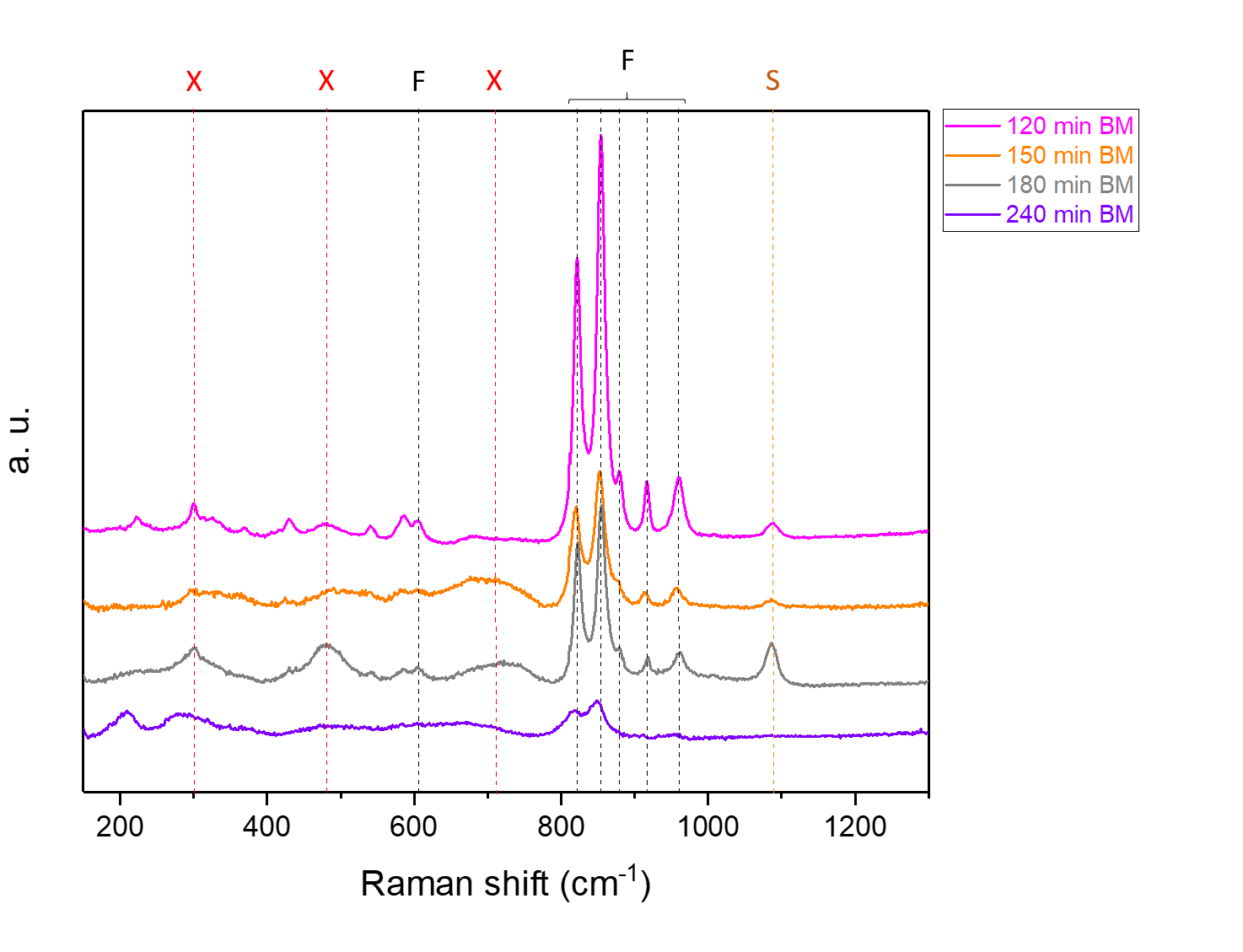
*

Figure S2: Enlargement of Figure 2: Raman spectra of the samples milled from 120 min to 240 min. Notes: F = Forsterite, X = hill-defined Fe(III) oxides, S = Siderite

^57^Fe Mössbauer spectroscopy analysis

Table S1: Summary of the relative area of each doublet plotted in Figure 4

| **Milling Time**  **(min)** | **Olivine site**  **M1** | **Olivine site**  **M2** | **Pyroxene**  **(Mg,Fe)Si_2_O_6_** | **Fe (III)**  **oxides** |
| --- | --- | --- | --- | --- |
|  | **Relative Area (%)** | | | |
| 0 | 0.47 ±.003 | 0.46 ±0.05 | 0.07 ±0.00 |  |
| 30 | 0.35 ±0.03 | 0.45 ±0.04 | 0.04 ±0.01 | 0.15 ±0.01 |
| 60 | 0.27 ±0.03 | 0.35 ±0.03 | 0.08 ±0.01 | 0.30 ±0.01 |
| 90 | 0.28 ±0.03 | 0.35 ±0.03 | 0.08 ±0.01 | 0.29 ±0.01 |
| 120 | 0.26 ±0.03 | 0.30 ±0.03 | 0.08 ±0.01 | 0.36 ±0.01 |
| 150 | 0.17 ±0.02 | 0.26 ±0.02 | 0.10 ±0.01 | 0.48 ±0.01 |
| 180 | 0.16 ±0.02 | 0.19 ±0.02 | 0.20 ±0.01 | 0.46 ±0.01 |
| 240 | 0.17 ±0.02 | 0.21 ±0.02 | 0.05 ±0.01 | 0.56 ±0.01 |





Figure S3: Plot of Gibbs free energy versus temperature for selected hydrogenation reactions, calculated by HSC Chemistry 6.

### References

1. Momma K., Izumi F., *J. Appl. Crystallogr.*, **44**, 1272 (2011).
